# Identification of important interacting proteins (IIPs) in *Plasmodium falciparum* using large-scale interaction network analysis and *in-silico* knock-out studies

**DOI:** 10.1186/s12936-015-0562-1

**Published:** 2015-02-08

**Authors:** Madhumita Bhattacharyya, Saikat Chakrabarti

**Affiliations:** Structural Biology and Bioinformatics Division, Council of Scientific and Industrial Research-Indian Institute of Chemical Biology, Kolkata, 700032 West Bengal India

**Keywords:** Centrality analysis, IIPs, *Plasmodium*, Graph theory, Host pathogen interaction, Hubs

## Abstract

**Background:**

*Plasmodium falciparum* causes the most severe form of malaria and affects 3.2 million people annually. Due to the increasing incidence of resistance to existing drugs, there is a growing need to discover new and more effective drugs against malaria. Despite the global importance of *P. falciparum*, vast majority of its proteins are uncharacterized experimentally. Application of newer approaches using several “omics” data has become successful for exploring the biological interactions underlying cellular processes. Till date not many system level study has been published using *P. falciparum* protein protein interaction. Hence, the purpose of this study is to develop a standardized pipeline for structural, functional, and topographical analysis of large scale protein protein interaction network (PPIN) in order to identify proteins important for network topology and integrity. Here, *P. falciparum* PPIN has been utilized as a model for better understanding of the molecular mechanisms of survival and pathogenesis of malaria parasite.

**Methods:**

Various graph theoretical approaches were implemented to identify highly interacting hub and central proteins that are crucial for network integrity. Further, potential network perturbing proteins via an *in-silico* knock-out (KO) analysis to isolate important interacting proteins (IIPs), which in principle, can elicit significant impact on the global and local environments of the *P. falciparum* interaction network.

**Results:**

177 hubs and 132 central proteins were identified from the malarial (proteins: 1607; interactions: 4750) PPI networks. Using the *in-silico* knock-out exercise 131 and 99 global and local network perturbing proteins were also identified. Finally, 271 proteins from *P. falciparum* were shortlisted as important interacting proteins (IIPs), which not only play crucial role in intra-pathogen network integrity, stage specificity but also interact with various human proteins involved in multiple metabolic pathways within the host cell. These IIPs could be used as potential drug targets in malarial research.

**Conclusion:**

Graph theoretical analysis of PPIN can be a very useful approach to identify proteins that are important for regulation of the interactions required for an organism’s survival. Important interacting proteins (IIPs) identified using *P. falciparum* PPIN provides a useful dataset containing probable candidates for future drug target analysis.

**Electronic supplementary material:**

The online version of this article (doi:10.1186/s12936-015-0562-1) contains supplementary material, which is available to authorized users.

## Background

Malaria is endemic to over 100 nations and territories in Africa, Asia, Latin America, the Middle East, and the South Pacific. *Plasmodium falciparum* transferred by a mosquito vector is by far the deadliest of the four human malarial parasite species. Though the intricate details of the pathogenesis are not yet clear, effective drugs against *P. falciparum* were in use since 1920. However, in present time traditional first-line treatments such as choloroquine and sulphadoxine/pyrimethamine have lost much of their effectiveness in many countries [1-3]. As a consequence new and more expensive anti-malarial drugs, including combination therapies, such as artemisinin-based combination therapy (ACT) were developed [4,5]. Development of a successful drug is highly dependent on the in-depth understanding of the organism’s biological processes. Exploring the protein-protein interactome of the parasite at the system level could be a useful strategy in unravelling its critical biological processes. New approaches like this will not only enhance the knowledge base about the underlying mechanism of parasite’s survival, but also will help us to identify proteins crucial for pathogenesis.

In the genomic and post-genomic era, increasing availability of genome and proteome information has led to the emergence of a new system biological approach where proteome level protein-protein interaction data is used for understanding an organism’s biology. In this approach PPINs or other biological networks are constructed and analysed to explore the organism specific structure and function of those networks [6-8]. Interestingly, these biological networks (*e.g*., protein-protein interaction, gene regulatory, signalling, and metabolic network) were found to follow the principles of graph and information theory [9,10]. According to graph theory a network’s compactness and capability of relaying information can be captured by the centrality analysis [9-12]. Network centrality indices reflect the nature of the network and node centrality indices reflect the property of the nodes. Node centrality indices are generally reflected via degree, closeness, radiality, betweenness, eccentricity, stress, wienner index, centroid, assortavity and clustering coefficient of the nodes whereas network centrality indices are usually represented by the average distance, connectivity, diameter, and clustering coefficient of the overall network [13-15].

It is generally observed that scale-free biological networks are robust towards random node removal and there are only few nodes in the network that are found to be crucial for network’s integrity [16-23]. Centrality calculation was important according to the centrality and lethality rule proposed by Albert L. Barabasi, which postulates that more central the protein is more lethal its removal could be for the network [24,25]. Hence, centrality analysis could lead to the identification of most important nodes for network integrity and subsequent perturbation of these important interacting proteins (IIPs) may lead to significant disruption of the network and/or the information flow through the network. In the last decade several studies were performed to explore, understand and establish the principles of network biology using biological network of different size and type [26-32]. The real time in vivo condition of a living cell was more closely reflected by these networks than investigating a cell’s physiology and function in small fraction by exploring interaction between two proteins or investigating a signalling pathway in great detail. Hence, in this study, PPIN from malaria parasite *P. falciparum,* a pathogenic apicomplexa, has been analysed to standardize a protocol for extracting nodes crucial for the network’s topological integrity as well as for the organism’s survival. Further, as a reference, similar analysis on PPIN extracted from the model non-pathogenic bacteria *Escherichia coli* has also been performed. In a scale-free protein-protein interaction network few proteins are connected with many neighbours where as other are connected with few [29,33,34]. These highly connected proteins are termed as hubs. Hubs were classified into many types based on the different approaches they were identified [35-38]. Here, hubs were classified into date and party hubs based on their spatiotemporal connectivity derived by their co-expression pattern [34,39]. In this study, a combined centrality score, termed as cumulative centrality score (CCS) was developed and all nodes were ranked according to their CCS. Proteins having significantly higher CCS than others were identified as central proteins (CP). An *in-silico* perturbation analysis of each node was performed and a node perturbation score was calculated measuring the network centrality parameters of the perturbed and unperturbed network. Perturbation potential of each node was estimated by the global network perturbation score (GNPS) as well as local network perturbation score (LNPS). Careful combination of these network parameters (hubness, centrality and perturbation potential) led to the identification of crucial nodes for the overall integrity of the PPIN. Finally, proteins that were found to be crucial for the PPIN as well as organism’s survival were considered to be most important and termed as important interacting proteins (IIPs). 271 and 220 proteins were identified as IIPs however, 16 and 19 proteins were found to be common in hubs, central and perturbing protein datasets in *Plasmodium* and *E. coli* PPIN, respectively. In *P. falciparum*, all of the 16 proteins were found to be part of core housekeeping proteome and involved in key homeostatic processes whereas nine among the 19 *E. coli* proteins were found to be essential genes. As new drug targets and mechanistic details of the parasite’s biology are still required, this kind of system level PPIN analysis could shed important insight towards better understanding of the complex life cycle of *Plasmodium*.

## Methods

### Construction of the network

Protein-protein interactions from *P. falciparum* (malaria network, MN) and *E. coli* (*E. coli* network, EN) with experimental evidences and high confidence scores [score > = 0.7] were extracted from the STRING database [40] and from a previous study [41]. Construction of MN and EN was validated by comparing them with the random networks generated by Barabasi-Albert (BA) preferential attachment algorithm [42,43]. For each biological network 10 random networks were created and average of the 10 network parameters were used for comparison. All the centrality parameters for the random networks are provided in Additional file 1: Table S1.

### Degree distribution

Degree distribution is an important indication of network architecture as scale free and random networks possess their distinctive degree distribution. Degree Distribution, *P(k)* of a network was defined as fraction of nodes in the network with degree *k*. If there are *N* nodes in total in a network and *n*_*k*_ of them have degree *k*, then

$$ P(k) = {n}_k/N $$

The degree distribution of random MN and EN networks were calculated using the above mentioned formula. The degree distribution of MN and EN followed power law (*P*(*k*) ~ *k*^−*γ*^ where γ is a constant) approximation whereas the degree distribution of the random networks were much smaller and followed the Poisson distribution. *f(k) = λ*^*k*^*e*^*–λ*^*/ k!* (where λ > 0) (see Additional file 2: Figure S1).

### Identification of hubs

Hubs were defined as proteins that have higher connectivity than others in the network. It was observed that hub proteins tend to be more important in network and were found to possess special biological properties [37]. The threshold degree to define a hub was set by two different and independent statistical approaches. In the first approach, all the degrees were normalized into z-score and the distribution was found to be positively skewed ranging from −0.6 to +12 for MN. The fraction of the degree population that contributes to this positive skew were extracted and separated. Rest of the population ranging from −0.6 to + 0.6 was found to have a normal symmetrical bell shaped distribution. The fraction of population degree having the z-score > = 1 was considered to possess significantly higher degree than rest of the population. In case of both the networks the lowest degree that has a z-score of 1 was 15. So, with this approach proteins having degree 15 or higher were considered as hubs (see Additional file 3: Figure S2A).

In the second approach, Mann–Whitney U test was performed to ensure if the threshold level was set correctly [44,45]. In the Mann–Whitney U test randomly 20 hubs and 20 non-hubs were selected at each of the degree threshold ranging from 5 to 20. Then the hubs and non-hubs were ranked based on their centrality scores. Based on this ranks, U value was calculated (formula mentioned below) and its significance was checked at 1% level. The whole process was repeated thousand times for each degree threshold. Finally, degree 15 was selected because hubs were found to be more central than non-hubs in more than 80% times at significance level 0.01with degree threshold of 15. This means that the nodes having degree 15 or higher are significantly different from nodes having degree lower than 15 in terms of their centrality (see Additional file 3: Figure S2B).

$$ U1=n1n2+\frac{n1\left(n1+1\right)}{2}-R1 $$

$$ U2=n1n2+\frac{n2\left(n2+1\right)}{2}-R2 $$

Where *U1* and *U2* are U value of sample 1 and sample 2; n1 and n2 are the sizes of sample 1 and sample 2; R1 and R2 are the sum of ranks of sample 1 and sample 2. The test statistic for the Mann–Whitney U Test is denoted as U and is the smaller of *U*_*1*_ and *U*_*2*_. The calculated U value is compared against a standard U table and two samples are considered significantly different when the calculated U value is smaller than the critical value of U.

### Identification of date and party hubs

Based on the spatiotemporal interaction pattern between the hubs and their interactors, hubs were classified as “date hubs” and “party hubs”. Hub interacting with all its neighbours at the same time and location were defined as party hub whereas hub that interacts with its neighbours at different time and location were defined as date hub [34]. Proteins interacting with each other at the same place and time are likely to be expressing together, hence co-expression analysis was implemented to identify the “date” and “party” hubs.

From different experiments eight expression profiles of *Plasmodium* genes were collected from PlasmoDB database [46]. Similarly, 11 expression profiles of *E. coli* genes were collected from GEO database [47]. Pearson’s correlation coefficient (PCC) of co expression between hub and its first level interactors were calculated for each dataset using the following formula [48].

$$ r=\frac{1}{\left(n-1\right)}{\displaystyle \sum_1^n\frac{\left(X-\mu X\right)\left(Y-\mu Y\right)}{\delta y\delta x}} $$

Where r is the Pearson’s correlation coefficient; *X* and *Y* are the values of two variables measured; *μX* and *μY* are the mean of X and Y; δ is standard deviations and n is the size of the sample.

Hubs with PCC > =0.5 were designated as party hubs and hubs with PCC <0.5 were considered as date hubs. 8 sets of date and party hubs were identified using 8 expression datasets. Finally, those hubs were selected for further analysis, which were commonly estimated as date or party hubs in 6 or more datasets (see Additional file 4: Table S2, Additional file 5: Table S3, Additional file 6: Table S4).

Topological overlap of nodes was estimated to validate the classification of hubs. A pair of nodes in a network is said to have high topological overlap if they are both strongly connected to the same group of nodes. All to all topological overlap (TO_ij_) matrix for 1607 nodes has been computed. Similarly, topological overlap of a module formed by a node X and all of its first level interactors were calculated using the following formula [49].

$$ T{O}_{ij}=\frac{{\displaystyle \sum_0^u{a}_{iu}{a}_{ju}}+{a}_{ij}}{\left( \min ki,kj\right)+1-{a}_{ij}} $$

Where α is the adjacency matrix value, *i* and *j* are the nodes for which TO is calculated, u is any other node, *ki* and *kj* are the degrees of node *i* and *j*.

$$ TOM=\frac{1}{N}{\displaystyle \sum_1^nT{O}_{ij}} $$

Where N is the number of interaction in each module and *TOM* is topological overlap of module.

### Analysis of functional similarity

Functional involvement of date and party hubs along with their interactors were investigated where each hub and its first level interactors (directly interacting) were regarded as a unit module and functional similarity between each hub and its interactors were checked using GO ontology [50].

*Plasmodium falciparum* proteins were annotated by homology based method. A BLASTp [51] search was done against the NCBI non-redundant (NR) sequence [52] and gene ontology (GO) database [50] using E-value filter < = 1e-05, query-coverage filter > = 50% and sequence identity filter > = 40%. Among the 1604 proteins forming the *Plasmodium* interaction network, 1030 proteins were annotated with biological function using the above mentioned homology approach. Fisher’s exact test [53] was performed to calculate the significance of GO term association to the MN proteins. All the associated GO terms were grouped into different categories and 21 categories were obtained for cellular component terms and 18 categories are obtained for biological process. For each 39 categories, 2x2 contingency table was constructed and Fisher’s exact P-value was calculated. For all the biological processes and cellular components P-value was observed to be lower than 0.01 validating that the association of GO terms were not by chance (see Additional file 7: Figure S3).

GO molecular function, molecular process and cellular compartmentalization of each hub and its first level interactors were extracted and compared. The similarity of GO ontologies among the hub and its interactors were calculated by matching the ontology keywords. The distribution of GO ontologies among the hub and its interactor proteins were represented in a percentage scale. Similarly, entropy and skewness of the GO ontology distribution within the hub and interactors were calculated using the following formulae.

$$ Entropy=-{\displaystyle \sum P\left({Y}_i\right) \log P\left({Y}_i\right)} $$

Where *Y*_*i*_ is information content of a random variable *Y* from a finite sample; P(Y_i_) is the probability mass function of *Y*_*i*_.

$$ Skewness=\frac{{\displaystyle \sum_{i=1}^N{\displaystyle {\left({Y}_i-\mu {Y}_i\right)}^3}}}{{\displaystyle {\left(N-1\right)\delta}^3}} $$

*μY*_*i*_ is mean of *Y*_*i*_; δis the standard deviation of *Y* and *N* is the sample size.

### Calculation of cumulative centrality score

Centrality values of the network were calculated to understand the topology and dynamics of the network. In this study 10 node centrality indices (degree, closeness, radiality, betweenness, eccentricity, stress, weinner index, centroid, assortavity and clustering coefficient) were calculated and four network centrality parameters (average distance, connectivity distribution, diameter and average clustering coefficient) were considered to measure the network centrality. The distribution of centrality parameters were shown as box whisker plot in Additional file 8: Figure S4.

Centrality values of each node were calculated using an in-house program. All the centrality values were normalized between 0 to1. A principal component analysis (PCA) was done (see Additional file 9: Figure S5) and three centrality parameters, betweenness, clustering coefficient and closeness were selected from the three selected principal components. Combined score (CS) was calculated by summing up the three parameters for each node. As a node’s centrality is heavily influenced by its neighborhood, a cumulative centrality score (CCS) was calculated by adding the CCS of a node and its directly connected neighbors. This CCS was considered as a measure of a node’s centrality. Global network centrality score (GNCS) was calculated as an average of CCS for the network.

$$ CS={\displaystyle \sum {C}_{Betweenness}+{C}_{Closeness}+{C}_{Clustering\_ coefficient}} $$

$$ CCS={\displaystyle \sum_1^nCS} $$

Where n is the Number of first degree interactors, *CS* is the combined score and *CCS* is the cumulative centrality score.

$$ LNCS=\frac{1}{N}{\displaystyle \sum_1^NCCS} $$

Where *LNCS* is the local network centrality score and N is the number of nodes in local sub graph.

$$ GNCS=\frac{1}{N}{\displaystyle \sum_1^NCCS} $$

Where *GNCS* is the global network centrality score and N is the number of nodes in the global network.

### Construction of local sub graph

For the creation of local sub graph, each protein having degree ≥ 2 were extracted along with its second level of interactors. For *P. falciparum,* 1,049 and for *E. coli,* 869 local sub graphs were formed. Clustering coefficient and network centrality score were calculated for each of the network. The topological viability of the local sub graphs was validated by linear relationship between clustering coefficient and LNCS. Non-radial connectivity pattern was indicated by positive values of both clustering coefficient and LNCS (see Additional file 10: Figure S6).

### Calculation of global and local network perturbation score

*In-silico* perturbation of the node was done by an in-house program, which sequentially removed single node and its interaction from the global as well as local (sub graph) networks. The consequence of a node’s removal was estimated on the integrity of the network and was measured by a network perturbation score (NPS). The network perturbation score (NPS) was calculated in two steps. In step one, NPS was simply measured by subtracting the global network centrality score (GNCS) of a network before and after perturbation of a particular node; higher the difference, higher the perturbation ability. Global and local perturbation score for each node *i* (GNPS_i_ and LNPS_i_) were calculated performing the perturbation in the global MN network and/or on the local sub graphs extracted via previously mentioned protocol. In step two, the perturbation score was re-ranked using the edge-weight considering the fact that a protein with higher average edge weight would be more impactful upon perturbation. To do this combined score (range 0.1 to 0.999) of interaction from STRING database was considered as edge weight and a combined edge-score for each node in MN was calculated using the following formula. This combined edge-score and network perturbation score (GNPS_i_ and LNPS_i_; calculated in step one) were combined by multiplication.

$$ {S}_{(x)}=1-{\displaystyle \prod_0^i1-{S}_i} $$

Where *S*_*X*_ is the combined edge score for node x, *i* is the number of interactor of node x. *S*_*i*_ is the STRING combined score for x-i interaction.

### Correlation of different scores

Correlation coefficient of z-scores of CCS, GNPS and LNPS of the same node were calculated to investigate the interdependence of the scores (see Additional file 11: Figure S7).

### Stage Specific interactions

Stage specific proteins were extracted from mRNA expression datasets [54,55]. The presence and absence of a gene was determined using the same protocol as reference 52. The proteins and their corresponding stages are mentioned in Additional file 12: Table S5. Expression levels of genes were normalized to 0 to 1 scale using the formula mentioned below.

$$ X\hbox{'}=\frac{X_i- \min (X)}{ \max (X)- \min (X)} $$

Where *X'* is the normalized value of *Xi* and min(*X*) and max(*X*) are minimum and maximum value of the population.

## Results and discussion

### Construction and validation of the PPI network

Protein-protein interactions from *P. falciparum* (malaria network, MN) and *E. coli* (*E. coli* network, EN) with experimental evidences and high confidence scores [score > = 0.7] were extracted from the STRING database [40] and from a previous study [41]. Construction of MN and EN was validated by comparing them with the random networks generated by Barabasi-Albert (BA) preferential attachment algorithm [43]. MN and EN were found to have scale free organization as their degree distribution followed power law. On the contrary both set of 10 corresponding random networks referred as malaria random networks (MRN 1 to 10) and *E. coli* random networks (ERN 1 to 10) showed binomial degree distribution. In Table 1 the topological properties of the MN and EN along with their randomized counterparts (MRN and ERN) are listed whereas the relative differences of various network properties are provided in Additional file 2: Figure S1, Additional file 8: Figure S4. The average clustering coefficients of the MN and EN were found to be quite low (0.12 and 0.07, respectively). High average degree, low clustering coefficient and low average distance of the PPINs denoted the radial pattern of interaction between hub and interacting partners.

**Table 1:** **Topological properties of** ***Plasmodium*** **and** ***E. coli*** **PPI Networks**

**Network parameters**	**MN**	**MRN**	**EN**	**ERN**
**No of nodes**	1607	1607	1505	1505
**No of edges**	4750	4750	4085	4085
**Average degree**	5.9	5.2	5.34	5.1
**Average shortest path**	4.39	4.5	4.14	4.5
**No of hubs**	177	612	126	526
**Degree threshold for defining hub**	15	11	15	9
**Average clustering coefficient**	0.12	0.001	0.07	0.006
**Max degree**	77	18	61	16
**Diameter**	12	9	14	9

### Identification and classification of hubs

**Figure 1:**
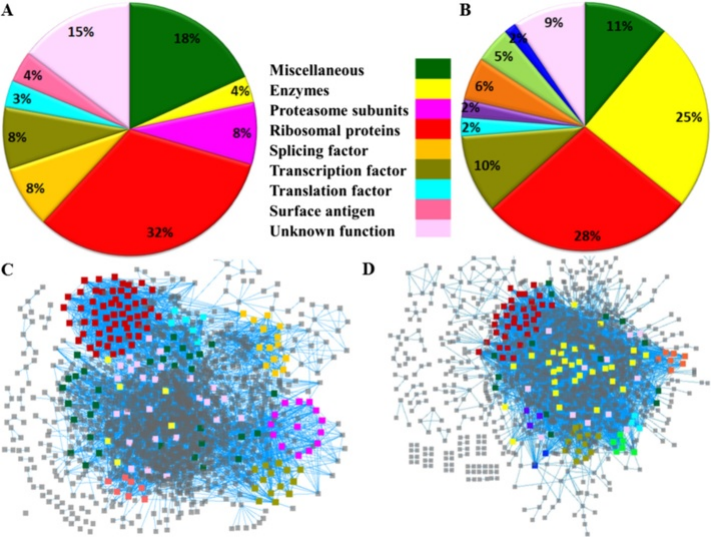
**Hub Proteins in MN and EN. (A-B)** Functions of hub proteins of MN and EN are plotted as pi-charts. Each function is highlighted in different colour. **(C-D)** Same hubs are highlighted on the network according to their function.

In a biological scale free network some proteins interact with many and some interact only with a very few partners. Hubs are proteins, which have higher degree (interaction) than others in the network, [30,33] thus may play crucial role in the regulation of network [33,34]. In this study, proteins interacting with more than 15 proteins were considered as hubs for both MN and EN. The degree threshold for defining a hub is determined by a rigorous two step statistical analysis (see Methods). In MN and EN, 177 and 126 proteins were identified as hubs. The functions of hubs were described in Figure 1 as pi-charts and the hubs are highlighted onto the network in different colour according to their biological function. Both the network possess non-modular dense connectivity pattern. The largest component contains 99% and 98% of the nodes in MN and EN, respectively.

**Figure 2:**
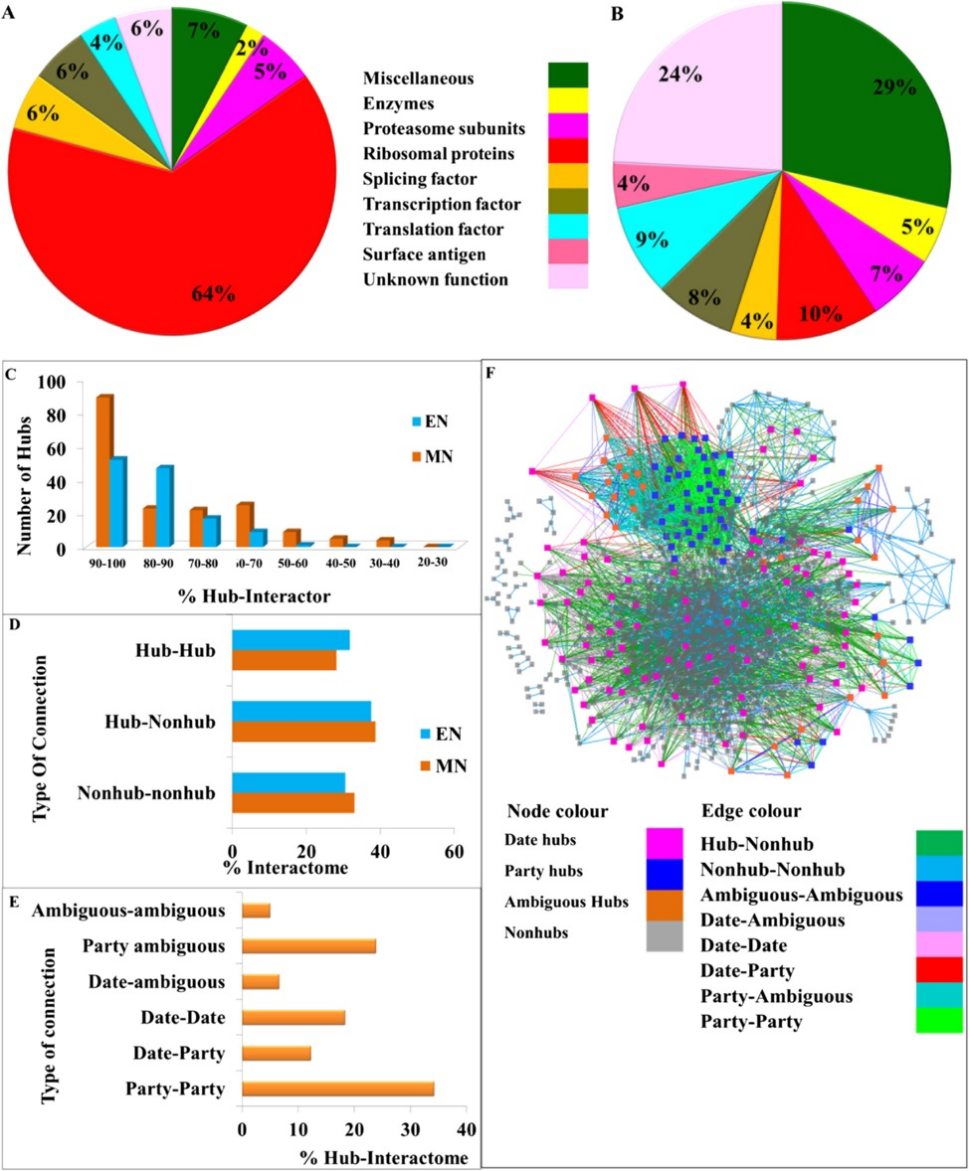
**Differential connectivity pattern of date and party hubs. (A-B)** Function of date and party hubs of MN are plotted as pi-chart. **(C-D)** Connectivity analysis of both MN and EN;%Hub-Interactor denotes the percentage of interactors which are also hubs;% Interactome denotes the percentage of total interaction. **(E)** Connectivity analysis of date and party hubs.% Hub-interactome denotes the percentage of core interactome contributed by hubs only. **(F)** Date and party hubs are highlighted on the network and their connections are also highlighted in different colour. Panel **E** and **F** suggest that party hubs are more connected to party hubs and date hubs are more connected to date hubs whereas date-party connections are comparatively lesser.

Based on the spatiotemporal interaction pattern between the hubs and their interactors, hubs were classified as “date hubs” and “party hubs”. Among the 177 hub proteins 52 hubs having the average Pearson correlation coefficient (PCC) of co-expression 0.5 or greater were selected as party hubs whereas 104 hubs with PCC value less than 0.5 were defined as date hubs (see Additional file 4: Table S2, Additional file 5: Table S3). For rest of the hubs date and party status were not certain hence those were termed as ambiguous hubs. Most of the party hubs were found to be ribosomal subunit (34) followed by RNA polymerase subunits (3), proteasome subunits (3), and splicing factors (3) along with miscellaneous proteins (4) including 3 proteins with unknown function. Date hubs showed a more varied functional involvement. Among the date hubs there were few ribosomal (9) and proteasome subunit (6) along with various other proteins like, enzymes (5), surface antigens (7), transcription factors and RNA polymerase subunits (8), translation factors (4), etc. (Figure 2A and 2B). In both the MN and EN all the hubs were connected and forming a core interactome of hubs surrounded by radially placed non-hub proteins (Figure 2C). Connectivity analysis revealed that in the MN, more than 66% interaction involved at-least one hub and 28% of interaction involved hubs as interacting pairs whereas in EN more than 69% interaction involved at-least one hub and 31% of interaction involved hubs as interacting pairs (Figure 2D). Both the networks were assortative in nature as hubs formed a densely connected core interactome (28% and 31% in MN and EN, respectively) whereas non-hub nodes were connected to hubs and resided at the periphery of the network. Even, date hubs were connected with more date hubs and party hubs were connected with party hubs (Figure 2E and 2F). On the contrary in case of EN though similar connectivity patterns among the hubs were observed yet no party hubs were found. In case of EN all the hubs have PCC of co-expression less than 0.5 (see Additional file 6: Table S4). This could be because of the lack of larger structural complexes like proteasome and spliceosome in *E. coli*. However, *E. coli* ribosomal subunits were also not found to be expressing in a correlated manner. Topological overlap score for each protein and its interactors were calculated and TOM or average topological over lap of a module (see Methods) was calculated for each hub and non hub protein. TOM scores for hubs were found to be much higher than nonhub proteins. Party hubs were found to have much higher topological overlap than date hubs validating the co-expression based classification of date and party hubs (see Additional file 13: Figure S8)

.

**Figure 3:**
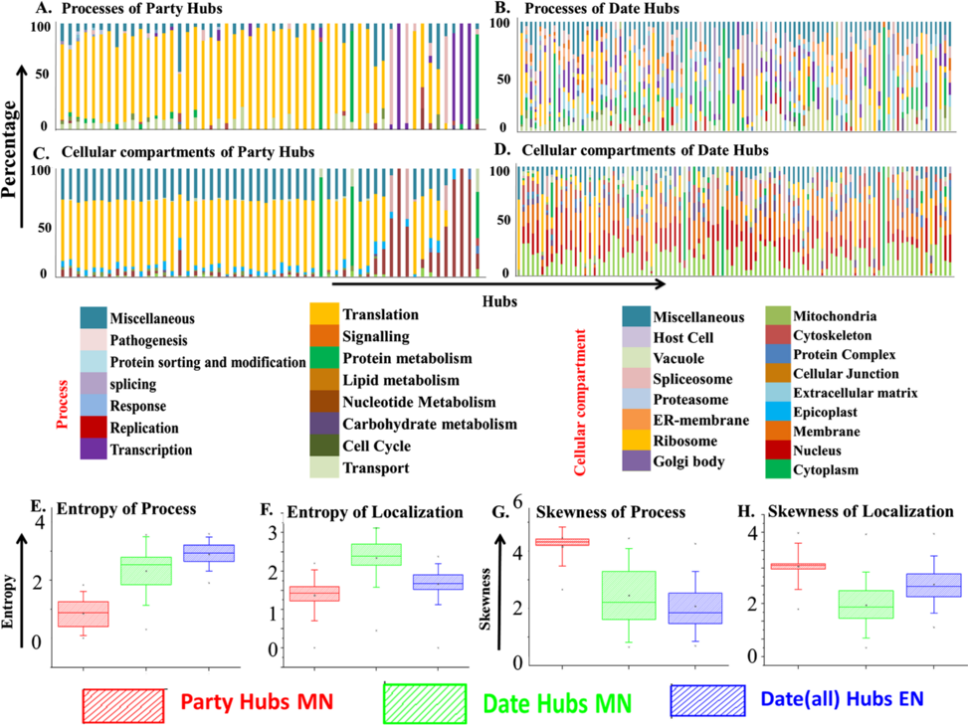
**Localization and functional similarities among date and party hubs. (A-D)** Similarity between hubs and their interactors in terms of their cellular localization and functional involvement. Each column represents the similarity of one hub and its interactors; their functional involvement and cellular localization are expressed as percentage along the Y axis; each colour on one column represents percentage of one cellular component or one biological process. **(E-H)** Box-whisker plots to show the differences in the skewness and entropy of the similarity function between the date and party hubs.

Functional involvement of date and party hubs along with their interactors were investigated where each hub and its first level interactors (directly interacting) were regarded as a unit module and functional similarity between each hub and its interactors were checked using GO ontology [50]. GO cellular compartment (C), molecular function (F) and molecular process (P) ontologies for each module were extracted and a similarity function for each module was calculated by comparing the GO ontologies among the hub and its interacting proteins. Distribution of fraction of proteins in each unit module involved in same ontology category was expressed by this similarity function (see Methods). Interestingly, no date hub was found to be involved in less than 5 GO processes whereas in case of all party hubs at least 50% of interactors were found to be involved in the same GO processes. Figure 3A shows that for all the party hubs, 50% of its ineractors are involved with a single GO processes such as translation, protein metabolism, transcription, and pathogenesis. Similar distribution of cellular components was also observed for party hubs and their interacting proteins (Figure 3C). On the other hand much more varied representation of cellular processes and localizations were observed for the identified date hubs and their interactors (Figure 3B and 3D). Further quantifications involving the types of processes and localization in terms of entropy and skewness suggest much higher entropy and lower skewness for the date hubs than those of party hubs (Figure 3E-3H).

### Identification of central proteins

Centrality values of the network were calculated to understand the topology and dynamics of the network. In this study 10 node centrality indices (degree, closeness, radiality, betweenness, eccentricity, stress, weinner index, centroid, assortavity and clustering coefficient) were calculated and four network centrality parameters (average distance, connectivity distribution, diameter and average clustering coefficient) were considered to measure the network centrality. The distribution of centrality parameters were shown as box whisker plot in Additional file 8: Figure S4. The distributions of centrality parameters for MN and EN were evidently different from that of their random versions (Additional file 8: Figure S4). In both PPINs, narrow range of clustering coefficient and low mean value of the same indicated the radial pattern of connectivity. Power law distribution of degree confirmed the scale free nature of this biological network. Narrow distribution of closeness and eccentricity also reconfirmed the assortative nature of MN network. The difference between a scale free network and a random network of same size were also distinctly evident in this plot.

**Figure 4:**
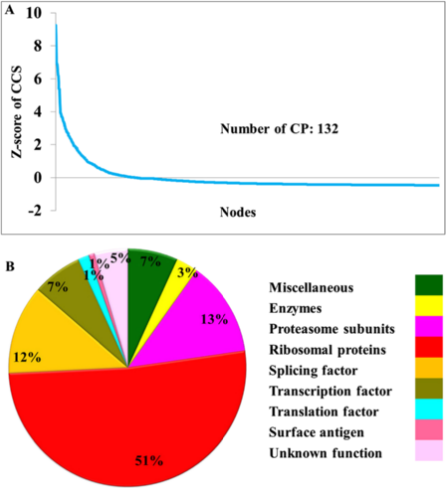
**Centrality analysis in MN. (A)** Distribution of global cumulative centrality score (CCS) of *Plasmodium* proteins normalized as z-score. **(B)** Distribution of different functions of the proteins belonging to CP-MN-132.

Two large matrix of 10 parameters for 1607 nodes (for MN) and 1505 nodes (for EN) ranging in different scale were created by the node centrality calculation. Using all these parameters a combined centrality score was calculated (see Methods) and normalized into 0 to 1 scale. The score was named as cumulative centrality score (CCS); higher the CCS more central the node is. All nodes in the network were ranked according to the CCS and nodes that have CCS significantly higher than others were extracted by a statistical z-score analysis. In MN and EN, 132 and 129 central proteins (CP) were found to have significantly higher CCS than others (Figure 4A and Additional file 14: Figure S9A). These two sets of central proteins were designated as CP-MN-132 and CP-EN-129 in MN and EN, respectively. Interestingly, not all CP were found to be hubs; 106 among 132 CPs are hubs while 32 and 53 are date and party hubs, respectively. Functions of proteins belonging to CP-MN-132 and CP-EN-129 sets were found to have similar kind of functions as plotted in Figure 4B and Additional file 14: Figure S9B. Apart from the node centrality score CCS, other network level centrality score such global network centrality score (GNCS) and local network centrality score (LNCS) (see Methods) were calculated and utilized in perturbation analysis.

### Identification of GNPP and LNPP

**Figure 5:**
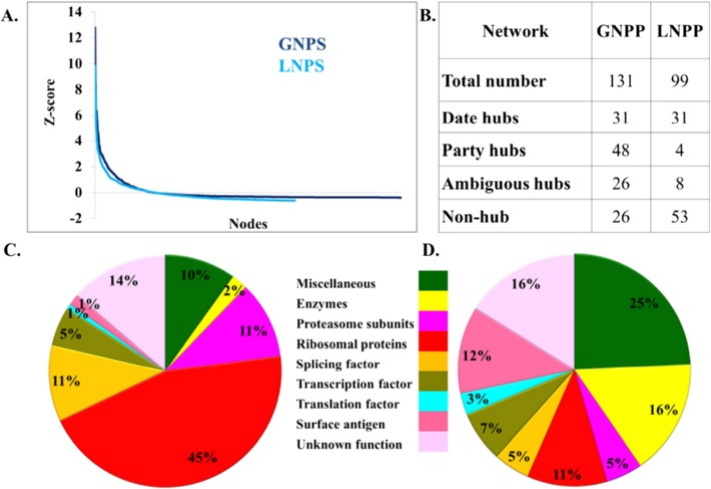
***In-silico*** **perturbation analysis in MN. (A)** Distribution of global and local network perturbation score (GNPS and LNPS) normalized as z-score in MN. **(B)** Fraction of hubs in GNPP-MN-131 LNPP-MN-99 data sets. **(C-D)** Distribution of different functions of proteins belonging to GNPP-MN-131 and LNPP-MN-99 protein sets.

An *in-silico* knock-out analysis was performed on the MN and EN to investigate the role of the crucial proteins in sustenance of the network integrity at the global and local sub graph level. A temporary local sub graph was created for each node considering the node and its 2^nd^ level interactors as a separate small network with the purpose of investigating perturbation effect of same node in global and local environment. The effect of node removal was measured by a global network perturbation score (GNPS), which reflects the change in network centrality before and after perturbation of a node from the network. The same scoring method was also applied in the local networks and local network perturbation scores (LNPS) were calculated. Proteins that have higher GNPS than others were identified by statistical z-score analysis (see Methods) and termed as global network perturbing proteins (GNPPs). In MN and EN 131 and 106 proteins were identified as GNPPs, respectively and were named as GNPP-MN-131 and GNPP-EN-106 (Figure 5A and Additional file 15: Figure S10A). In GNPP-MN-131, 99 proteins were found to be hubs. Functions of proteins of both GNPP-MN-131 and GNPP-EN-106 were plotted as pi-charts in Figure 5C and Additional file 15: Figure S10C.

A local network perturbation score (LNPS) was calculated for 1049 proteins in MN and 875 proteins in EN. Proteins that have higher LNPS than others were identified by statistical z-score analysis (see Methods) and termed as local network perturbing proteins (LNPPs). In MN and EN 99 and 91 proteins were identified as LNPPs, respectively and were named as LNPP-MN-99 and LNPP-EN-91 (Figure 5A and Additional file 15: Figure S10A). Functions of proteins of both GNPP-MN-131 and GNPP-EN-106 were plotted as pi-charts in Figure 5D and Additional file 15: Figure S10D.

From the above experiments it was observed that party hubs were more central than date hubs. The effect of perturbation when measured in global network, was almost same for party and date hubs but in local subgraphs date hubs showed much higher perturbation effect than the party hubs (see Additional file 16: Figure S11).

### Identification of important interacting proteins (IIPs)

**Figure 6:**
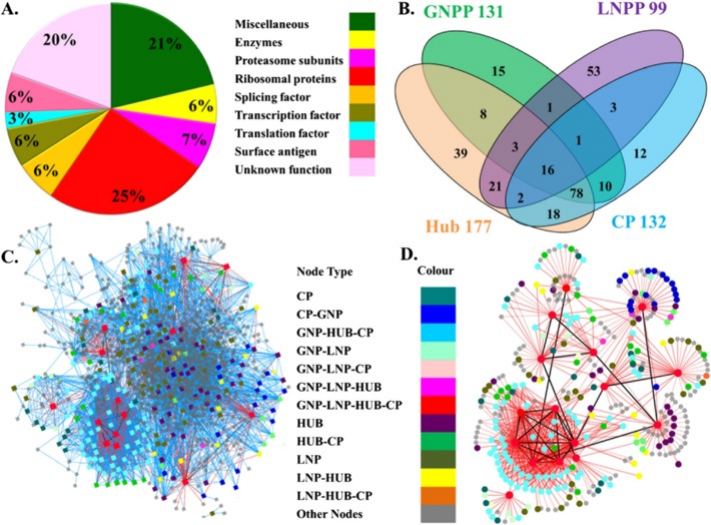
**Identification of important interacting proteins (IIPs) of MN. (A)** Distribution of different functions in 271 proteins important for network topology. **(B)** Overlap between the selected proteins by different methods is presented in the Venn diagram. **(C)** 271 important interacting proteins are highlighted according to their selection by different methods. **(D)** Interaction network of MN-16. In **C** and **D** panels, these16 proteins are highlighted in red colour and interactions among themselves are denoted by black lines. Interaction among these 16 proteins and other proteins of IIP-MN-271 are denoted by red lines. Rest of the interactions among IIP-MN-271 proteins and other nodes are denoted by blue lines.

So far, we described how proteins important for network integrity were identified from various independent perspectives. Next, the scores (CCS, GNPS and LNPS) of each protein were compared to investigate the relationship among the scores. The CCS and GNPS have a correlation coefficient of 0.7 but the LNPS is not correlated with any of them (see Additional file 11: Figure S7). Hubs were proteins with degree 15 and above having higher connectivity than other nodes in the network, CPs were proteins central to the network, whereas GNPPs and LNPPs were proteins which elicited measurable perturbation effect on global and local network environments, respectively. These four sets of proteins tagged as HUB, CP, GNPP, and LNPP were overlapping (Figure 6B and Additional file 17: Figure S12B); hence a total number of 271 and 220 unique proteins were identified in MN and EN that were present at least in one of the four sets. These protein sets were termed as IIP-MN-271 and IIP-EN-220. Almost 80% and 90% of these proteins from MN and EN have some known functional relevance. Similarly, large fractions (75% and 74%) of the total interactions in the MN and EN were contributed by these 271 and 220 proteins. Thus a highly connected core interactome was constituted by these 271 and 220 proteins in both MN and EN (Figure 6C and Additional file 17: Figure S12C). Details of these IIP-MN-271 proteins are provided in DatasetS1 (see Additional file 18: Dataset S1), which is a database for malarial important interacting proteins [56]. However, only 16 and 19 proteins were extracted from these 271 MN and 220 EN proteins, which belonged to the all four constituent set (*i.e*., HUB, CP, GNPP and LNPP). These proteins are termed as MN-16 and EN-19.

These 16 proteins are involved in 515 interactions with 318 other proteins which as a whole constituted a significant fraction of the network (12%) (Figure 6D). Interestingly, these proteins were found to be the most important housekeeping proteins and part of the central homeostatic process. There are three proteasome subunits among which two have endopeptidase activity and one is a regulatory subunit. Seven ribosomal subunits were also present, among which three are part of large subunit, three are part of small subunit and one is part of large subunit of mitochondrial ribosome. Among these proteins, three proteins were identified which have no homologues in human and possess virulent properties. These three proteins are PF10_0232 - a chromodomain helicase protein, PFI1475w – a merozoite surface protein (MSP1), PF13_0228 - a 40S ribosomal subunit. PF10_0032 has similarities with virulence proteins from *Candida albicans* and *Vibreo cholerae*. This ATP dependent helicase protein is located in nuclear chromatin and involved in nucleosome assembly and regulation during chromatin remodelling. PF10_0032 interacts with 57 other proteins which include replication factors, surface antigens like ETRAMP 7.5 and MSP-1,7,9, ubiquitin ligase, DNA binding chaperones, transcription factor, other helicase and many conserved protein with unknown function. PFI1475w - merozoite surface protein 1 is a GPI anchored membrane protein and part of erythrocyte invasion machinery. This well-known virulence factor had 51 interacting partners including apicoplast ribosomal protein and DnaJ protein, QA-SNARE protein, transcription factors, secretory protein, nuclease, other MSPs, response proteins, calmodulin, ubiquitin ligase, chromosome maintenance, proteasome subunits, and many conserved *Plasmodium* protein with unknown function. PF13_0228 is a protein of small subunit of 40S ribosome and interacts with 42 proteins which include E3 ubiquitin ligase, chromodomain helicase, rhoptry neck protein, serine protease and esterase, RNA methyltransferase, erythrocyte binding protein, liver stage antigen, RNA polymerase I, AAA family ATPase, chromosome associated protein along with many other ribosomal subunit.

On the other hand, 19 proteins of EN-19 set had a total of 743 interactions with 380 (24%) number of partners which as a whole constituted 18% of the network (Additional file 17: Figure S12D). Interestingly, these proteins also were found to be the most important housekeeping proteins and part of the central homeostatic process of *E. coli*. Nine among these 19 proteins were found to be essential for *E. coli*. All the proteins in MN-16 and EN-19 resided in the top 100 bin when their PageRank [57] were calculated and analysed. Detailed information about MN-16 and EN-19 are listed in Additional file 19: Table S6, Additional file 20: Table S7.

### Stage specific networks

**Figure 7:**
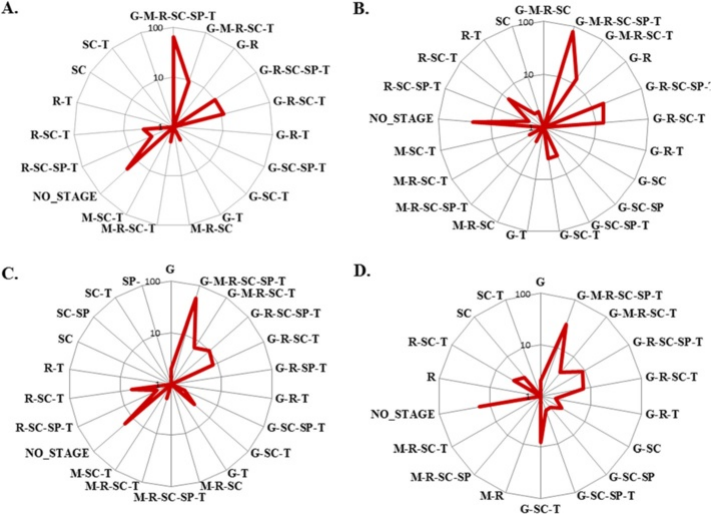
**Distribution of important proteins across different life cycle stages.** In this diagram, stage specific expression pattern of proteins from Hub **(A)**, CP **(B)**, GNPP **(C)** and LNPP **(D)** set are presented as a radar chart. In all the panels, stage(s) are plotted as single points at the periphery where G stands for gametocyte, SP stands for sporozoite, SC stands for schizont, T stands for trophozoite, M stands for merozoite and R stands for ring stage. Numbers of proteins from each of the stage(s) are plotted along the Y axes.

As intra human life cycle stages of *P. falciparum* occur at different host tissues it will not be irrational to expect involvement of different sets of proteins to create a stage specific PPIN. Hence, stage specific proteins along with their PPI were extracted for six intra-human stages such as sporozoite, merozoite, trophozoite, schizont, ring stage and gametocyte [54,55]. Only those interactions were considered as stage-specific where both the interacting partners were expressed in the same stage. Total 3,598 interactions among 1,507 proteins were found where both the partners were present (expressing) in the same stage. Apart from 315 interactions which were unique to any of the six stages all the other interaction were overlapping among two to six stages. The number of nodes and edges present in each stage were mentioned in Table 2. Stage specific expression pattern of IIP-MN-271 proteins can be viewed in DatasetS1 [56]. Among the MN-16 proteins 7 were present in all stages, PF13_228 and PF10_111 were absent in merozoite stage, PF11_0303 was absent in merozoite and sporozoite stage whereas PF10_0038 was absent in gametocyte, merozoite and sporozoite stage. Presence of hubs, CPs, GNPPs and LNPPs were investigated across different life cycle stages. These important proteins were distributed evenly in all life cycle stages (Figure 7). For all of these six life cycle stages, six unique networks were constructed and analysed. Average centrality values of these networks are presented in Additional file 21: Table S10. Average network centrality values of these are quite similar reason of which may be presence of a common core of interactions in all of them, However, the networks were compares among themselves and a wide range of interactions were found to be overlapping among them (see Additional file 22: Figure S13).

**Table 2:** **Number of proteins and interactions in different life cycle stages of** ***Plasmodium falciparum***

**Name of stage**	**For MN**			**For MN-14**		
	**(Node:1605, Interaction:4750)**			**(Node:303, Interaction:523)**		
	**Number of stage specific interaction**	**Number of stage specific node**	**Number of unique interaction**	**Number of stage specific interaction**	**Number of stage specific nodes**	**Number of unique interaction**
**Sporozoite**	1458	617	9	248	140	2
**Merozoite**	1155	438	1	190	100	0
**Trophozoite**	3074	1126	66	378	218	1
**Ring stage**	2638	909	35	364	204	2
**Schizont**	3079	1132	99	388	225	4
**Gametocyte**	2635	1062	105	334	201	7

### Host interacting proteins

**Figure 8:**
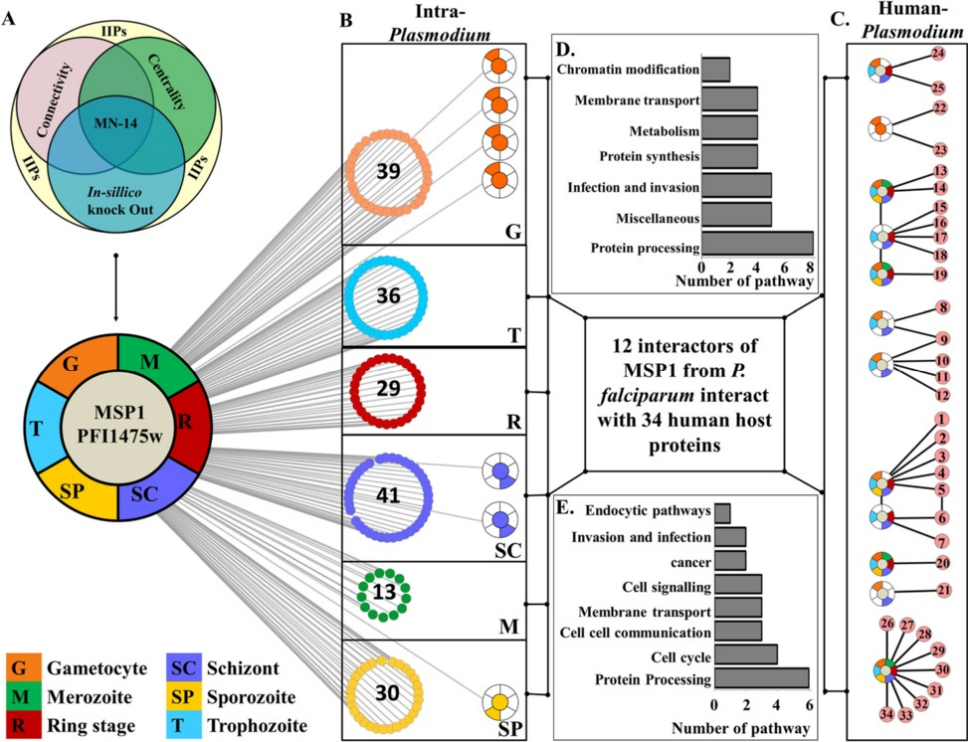
**A schematic diagram of MSP1 and its interactions in** ***Plasmodium*** **and human. A** A schematic Venn diagram for identification of important proteins. PFI1475w (MSP1) is one of the IIPs identified using this protocol and presented here as an example. In the panel **B** intra *Plasmodium* stage specific interactions of MSP1 are represented as schematic circular networks in six different boxes where each box represent a particular life cycle stage marked at the bottom right corner of the box. Unique stage specific interactors are marked separately in the boxes. In panel **C** human partners of these MSP1 interacting proteins are presented. The numbers on the proteins corresponds to the name mentioned at Additional file 23: Table S8. Pathways of the *Plasmodium* interactors (from panel **B**) and human proteins (from panel **C**) are represented as two bar charts (panel **D** and **E**, respectively). The proteins are represented here as small circles and their corresponding stages are represented as a circular colour pattern at the circumference of the circle. The stage(s) where the particular protein is expressed is filled with corresponding colour and other stage(s) are left blank.

Among the 1604 proteins in the MN network, 152 were found to interact with human host proteins. All these interactions were established by an inter-species yeast two hybrid assay [58]. Among these 152 proteins, 35 were found to be part of the 282 important interacting proteins for the MN network. These 35 proteins interact with 91 human and 351 *Plasmodium* proteins forming a total 644 interactions (Table 3). Among these 91 human partners 39 were mapped onto 65 KEGG [59] pathways including signalling pathways (8), infection mechanism (11) and metabolic pathways (6) as the most frequent ones. Among the signalling pathways Hedgehog signalling, NOD signalling, MAPK signalling, and TOLL-like receptor signalling pathways were found to contain at least one protein that interacts with one or more *Plasmodium* proteins. Similarly, pathways involved in general infection (*e.g*., bacterial infection, toxoplasmosis, trypanosomiasis and viral infection) and cellular communication (*e.g*., endocytosis, phagocytosis, cell-cell adhesion, and tight junctions) were also found to be affected by these host interacting proteins from *P. falciparum*. Malfunction of these pathways might result into characteristic clinical manifestations of malaria (see Additional file 23: Table S8). Host interactions of MN-16 proteins were investigated separately. All the 16 proteins were found to have no direct host connection but their 1^st^ level interactors had direct interaction with many human proteins. In Figure 8 such a scenario is described using PFI1475w (MSP1) as an example. PFI1475w, which is expressed in all life cycle stages of *Plasmodium* interacts with different proteins in different stages creating a dynamic interaction pattern across the life cycle. 12 among these 51 interactors were found to interact with 34 human proteins which in turn were part of 22 different pathways. Detailed information about MSP1 and other proteins were described in Additional file 24: Table S9.

**Table 3:** **Number of** ***Plasmodium*** **proteins that interact with human proteins**

	**Number of interactors in Human**	**Number of interaction between Human and** ***Plasmodium*** **proteins**	**Number of interactor in** ***Plasmodium*** **(MN)**	**Number of intra-pathogen interaction**
***Plasmodium*** **proteins having host partners152**	257	367	515	996
**IIP-271 having interacting host partner 35**	77	103	351	541

## Conclusion

The search of an effective method to identify important protein(s) within a network was started since two decades ago but only a few centrality based methods were reported [26,32]. However, due to the heterogeneous structure and organization of different networks no generic method could be established. Here, in this study we made an attempt to establish a protocol for finding proteins that are crucial for PPI network topology. Incorporation of biologically rational filtration system further led us to identify proteins, which could be crucial for an organism’s survival. In case of *P. falciparum,* 16 proteins were identified, among which three have the potential to be therapeutic targets. The gene essentiality index for *P. falciparum* is not available but identification of similar housekeeping enzymes as IIPs in *E. coli* indicated that this method could actually identify set of proteins, which are important for an organism’s survival. The importance of the IIPs was again validated when they were compared with PageRank of the nodes in both of the network [57]. PageRank is an algorithm generally used for finding important websites in the internet, a giant scale free network. All the proteins in both MN-16 and EN-19 were suggested to be within the top 100 ranks indicating that these nodes are important for the connectivity and flow of information through the corresponding PPI network. Identification of date and party hubs is important and all the date hubs in the *Plasmodium* network were connected and a long chain of hubs were formed. A heavily connected core interactome of hubs was observed in these networks where hubs were connected more with each other than being connected to non hubs. Interestingly, although both the networks (MN and EN) were observed to be scale free yet none of them possess modular architecture like the yeast PPIN [34,60-62]. Absence of modular architecture in both the organisms and absence of party hubs in *E. coli* indicated that the PPIN of different organisms might have different architecture and connectivity. However, none of the interaction network was complete enough to draw a conclusion about its architecture as these large scale proteome analysis experiments could not capture more than 25% to 30% of the whole proteome. The actual interaction pattern will be established only when all the PPI of an organism could be captured and assembled. In this study, crucial proteins were identified from four different independent perspectives and then combined together to identify proteins that are important for the overall integrity of the organisms’ interactome. Combination of all the centrality parameters was critical to find out truly central proteins. Interestingly all the MN-16 proteins were found to be part of homeostatic pathways, which are minimal for an organisms survival indicating that these proteins could be part of the primordial protein set for the organism. Extraction of stage specific interactions makes it evident that proteins of *Plasmodium* interacts with different partners at different stages and generates a dynamic PPIN. There is a future scope to investigate this interaction dynamics for better understanding of *P. falciparum* biology. Our protocol was standardised on the intra pathogen PPIN to identify the IIPs but this can be practically applied over any PPIN as well. Further, interacting partners of the parasitic IIPs were found within the human cell and shown that the human interactors mostly act as crosstalk protein among various metabolic, signalling and disease pathways. This in turn establishes the importance of IIPs in *Plasmodium* life cycle. However, to get a better idea about the influence of the parasitic proteins within the host cell, future study should be concentrated where the tripartite host pathogen interaction network comprising of (i) interaction among parasitic proteins, (ii) interaction among host proteins and (iii) interaction among host and pathogen proteins can be constructed and subsequently analysed.

## Additional files

Additional file 1: Table S1. Topological Properties of random network. In this table all the network centrality parameters of randomized networks are generated. Additional file 2: Figure S1. Degree distribution of MN and EN along with their random counterparts MRN(1–10) and ERN(1–10). Degree distribution of MN and EN is distinctly different from the random networks as random networks follow binomial degree distribution while MN and EN follow a power law degree distribution. Additional file 3: Figure S2. Identification of Hubs. In this approach (1) for determination of degree threshold for hub, z-score of degree is plotted as a positively skewed normal distribution having z-score at X axis and probability density function of z-score at the Y axis. Green lines denote mean median and mode if the fraction of the distribution that contributes to the skewness of the graph is omitted, the rest of the distribution turns out to be standard normal with mean median and mode (green line) at 0 (inset). The z-score threshold after which the distribution becomes skewed is 0.7; so z-score of 1 which correspond to degree 15 is considered as the threshold z-score for hub definition **(A)**. Approach 2 for Hub identification is described as a flow chart **(B)**. Additional file 4: Table S2. Date hubs of *Plasmodium falciparum.* In this table date hubs of MN are mentioned along with their name and correlation coefficient. Additional file 5: Table S3. Party hubs of *Plasmodium falciparum.* In this table party hubs of MN are mentioned along with their name and correlation coefficient. Additional file 6: Table S4. Date hubs of *E. coli.* In this table date hubs of EN are mentioned along with their name and correlation coefficient. Additional file 7: Figure S3. Distribution of Fisher exact P-value for GO categories. Fisher’s exact P-value has been calculated for 18 biological process (green) and 21 cellular component (red) categories using 2x2 contingency table. Additional file 8: Figure S4. Distribution of centrality parameters for MN and EN along with their random counterparts shown as box whisker plots. Boxes are 2^nd^ and 3^rd^ percentile of the distribution and whiskers are standard deviations. Mean is presented by a small square on the distribution and median is a straight line on the boxes. Maximum and minimum values are denoted by a ‘X’ sign. For all plots P-value MN and MRN are less than 0.01 indicating the significance of difference between the centrality parameters of MN and MRN. P-value for EN and ERN is also less than 0.01 in all cases indicating that *E. coli* PPINs are essentially different from the ERNs. Additional file 9: Figure S5. Principal component analysis. All (9) centrality parameters were normalized to 0 to 1 scale and an all-to-all correlation matrix was constructed. Three principal components were identified and one parameter from each component was selected (highlighted by blue rectangle) for further calculation of combined centrality score. Additional file 10: Figure S6. Relationship between LNCS and clustering coefficient. The plot is generated for 1049 local sub graphs created from MN. From the proportional increase of local network centrality score to clustering coefficient it is clearly evident that the constructed sub graphs are not randomly connected rather they have a non-radial connectivity like scale free networks. This also validates that the network centrality score described in this article is a true indicator of centrality. Additional file 11: Figure S7. Relationship of CCS, GNPS and LNPS. Centrality score and global network perturbation score are correlated but none of them is correlated with local network perturbation score. Additional file 12: Table S5. Normalized expression profile of *Plasmodium* Proteins in different life cycle stages. This is the expression profile of *Plasmodium* Proteins in 6 life cycle stages. Additional file 13: Figure S8. Topological overlap of hub and non hub modules. Distribution of TOM scores of date hubs, party hubs and non hub proteins. Additional file 14: Figure S9. Centrality analysis in EN. Distribution of global cumulative centrality score (CCS) of *E. coli* proteins normalized as z-score **(A)**. Distribution of different functions of proteins belonging to CP-EN-121**(B)**. Additional file 15: Figure S10.
*In-silico* perturbation analysis in EN. Distribution of global and local network perturbation scores (GNPS and LNPS) normalized as z-score in EN **(A)**. Fraction of hubs in GNPP-EN-106 and LNPP-EN-91 data sets **(B)**. Distribution of different functions of proteins belonging to GNPP-EN-106 and LNPP-EN-91 protein sets **(C-D)**. Additional file 16: Figure S11. Differential perturbation effect of date and party hubs in global and local networks. Distribution of perturbation scores (normalized as Z-score) of party and date hubs in global and local networks. Correlation coefficient of GNPS and LNPS of is 0.4 for party hubs and 0.1 for date hubs **(A-B)**. Distribution of perturbation scores of party and date hubs for global and local networks shown as box whisker plots **(C-D)**. Additional file 17: Figure S12. Identification of important interacting proteins (IIPs) of EN. Distribution of different functions in 220 proteins important for network topology **(A)**. Overlap between the selected proteins by different methods is presented in the Venn diagram **(B)**. 220 important proteins are highlighted according to their selection by different methods **(C)**. Interaction network of EN-19 **(D)**. In C and D panels, EN-19 proteins are highlighted in red colour and interactions among themselves are denoted by black lines. Interactions among these 19 proteins and other proteins of EN-220 are denoted by red lines. Rest of the interactions among EN-220 proteins and other nodes are denoted by blue lines. Additional file 18: Dataset S1. MIIP: Malarial Important interacting Proteins. This is a brief description of MIIP database. Additional file 19: Table S6. Details of MN-16. In this table the topological and functional details of MN-16 proteins are provided. Additional file 20: Table S7. Details of EN-19. In this table the topological and functional details of EN-19 proteins are provided. Additional file 21: Table S10. Average Network Centrality parameters of 6 stage specific Networks. For each of the six life cycle stages six unique PPI network has been constructed analysed using graph theory principle. The table contains the average network centrality parameters for each of the stage. Additional file 22: Figure S13. Pairwise overlap of interactions among six life cycle stages. Pairwise overlaps of interactions among six life cycle stages are presented in the colour matrix. Six stages are represented by 6 colours as indicated in the name of the stage. The central hexagon (grey) indicates the common core among all stages and the first block on the arms of hexagon (grey in colour) contains the total number of unique interactions. The other consecutive blocks represent the overlapping interaction with other 5 stages represented by blocks of corresponding colour. Additional file 23: Table S8. Pathway details of Human and *Plasmodium* Proteins. In this table 35 IIPs which have interaction with human proteins are reported along with their human partners along with their pathways. Additional file 24: Table S9. Interaction details of PFI1475w (MSP1) in *Plasmodium* and human. This table shows the complex interaction pattern of a virulent *Plasmodium* protein and its corresponding human interactions.

## Abbreviations

PPIN: Protein protein interaction network
MN: Malaria network
EN: *E. coli* Network
PCC: Pearson correlation coefficient
CCS: Cumulative centrality score
GNCS: Global network centrality score
LNCS: Local network centrality Score
CP: Central proteins
GNPP: Global network perturbing proteins
LNPP: Local network perturbing proteins
IIP: Important interacting proteins
