# Supplementary figures and images for: Identification of important interacting proteins (IIPs) in Plasmodium falciparum using large-scale interaction network analysis and in-silico knock-out studies
Source: Malar J. 2015 Feb 8;14:70. doi: 10.1186/s12936-015-0562-1 (PMC4333160; doi:10.1186/s12936-015-0562-1)

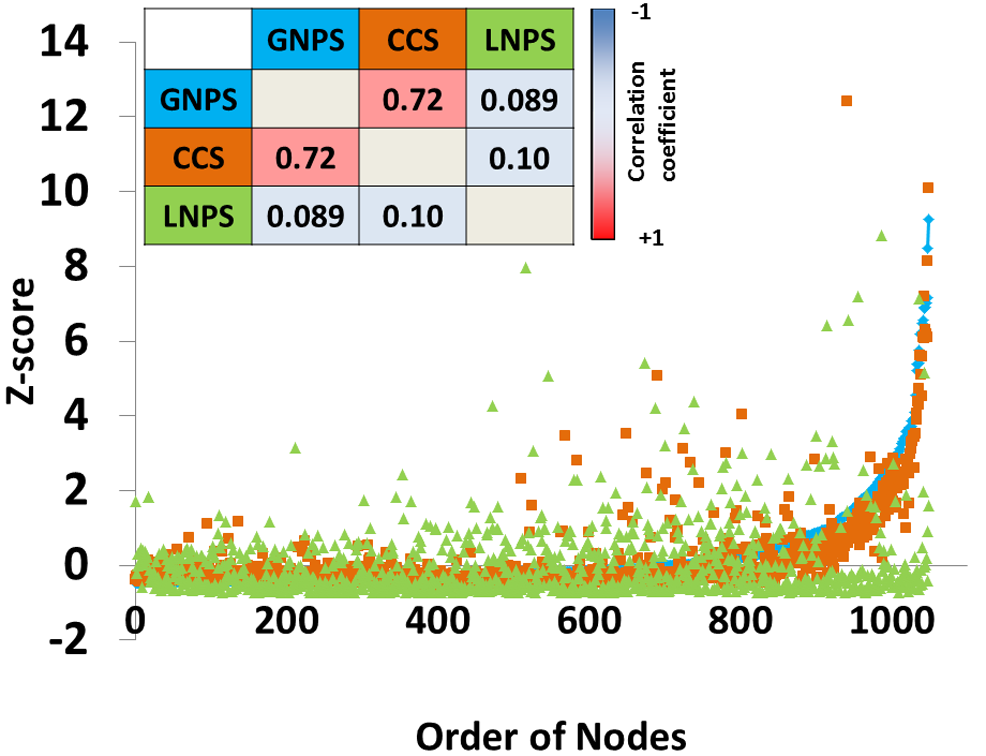

Supplement: Additional file 11: Figure S7. — Relationship of CCS, GNPS and LNPS. Centrality score and global network perturbation score are correlated but none of them is correlated with local network perturbation score. [file 12936_2015_562_MOESM11_ESM.tiff]

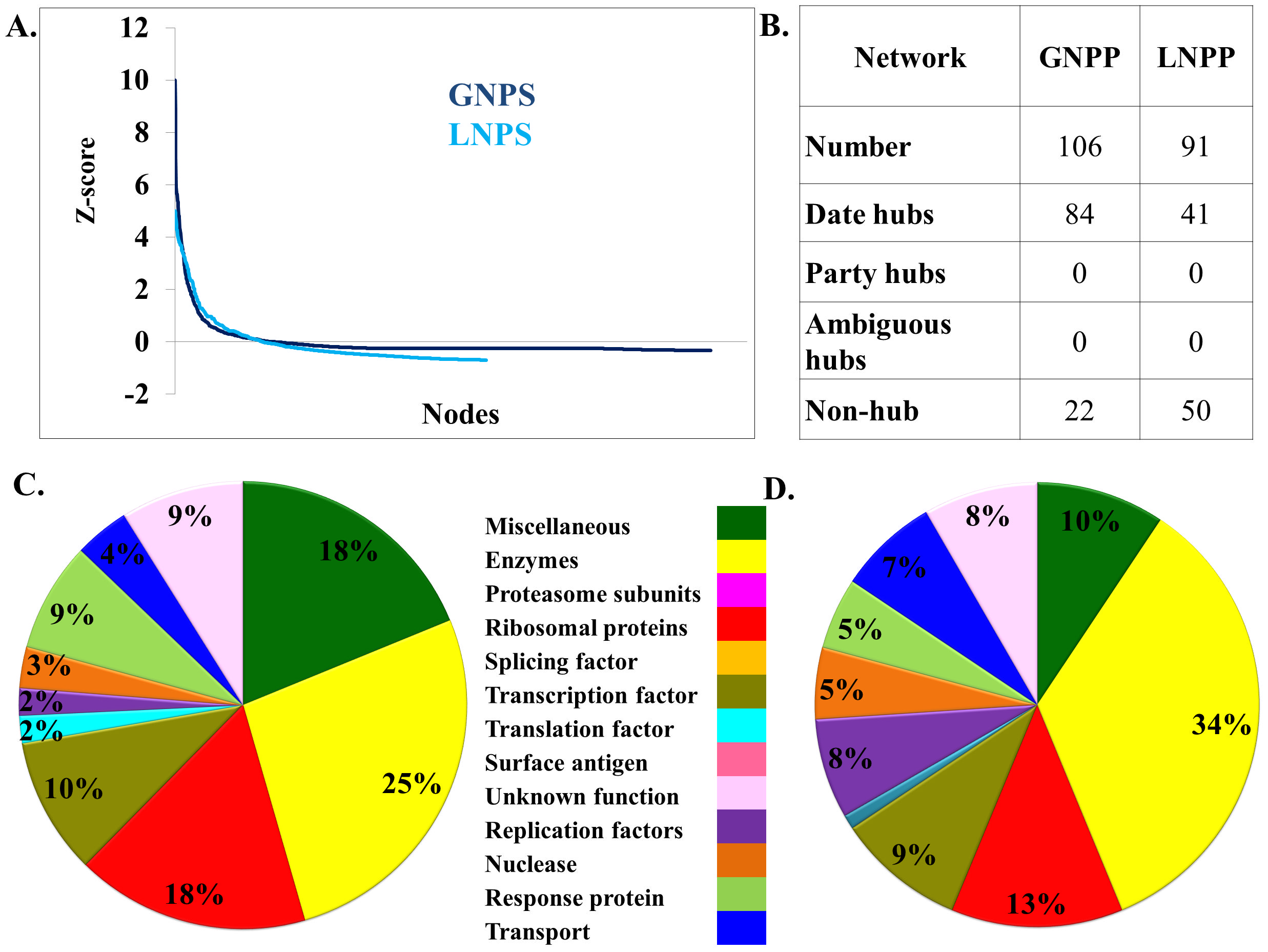

Supplement: Additional file 15: Figure S10. — In-silico perturbation analysis in EN. Distribution of global and local network perturbation scores (GNPS and LNPS) normalized as z-score in EN (A). Fraction of hubs in GNPP-EN-106 and LNPP-EN-91 data sets (B). Distribution of different functions of proteins belonging to GNPP-EN-106 and LNPP-EN-91 protein sets (C-D). [file 12936_2015_562_MOESM15_ESM.tiff]
