# Supplementary material for: Identification of important interacting proteins (IIPs) in Plasmodium falciparum using large-scale interaction network analysis and in-silico knock-out studies
Source: Malar J. 2015 Feb 8;14:70. doi: 10.1186/s12936-015-0562-1 (PMC4333160; doi:10.1186/s12936-015-0562-1)
Supplement: Additional file 18: Dataset S1. — MIIP: Malarial Important interacting Proteins. This is a brief description of MIIP database. [file 12936_2015_562_MOESM18_ESM.doc]

MIIP

Important interacting proteins (IIPs) are key regulators of a protein-protein interaction network (PPIN) and play crucial role in network topology and function. IIPs are identified from *Plasmodium falciparum* PPIN by combination of multiple independent approaches such as connectivity analysis, centrality analysis and *in-silico* perturbation analysis. A new and useful protocol has been developed for identification of IIPs using the combined analysis. The protocol has been developed as a package called NetAnPf which could be implemented on any PPIN for identification of IIPs.

MIIP is a database for 271 malarial IIPs, which are classified into 4 sets named as HUB, CP, GNPP and LNPP. In the database we provide the details of 282 proteins. Along with that unique stage specific interactions are also presented here as separate tables. Host interactions of 282 proteins are also provided. Finally in this website a download link for the developed NetAnPf package is also provided.

URL for the database: http://www.hpppi.iicb.res.in/pfnet/
