# Supplementary material for: Identification of important interacting proteins (IIPs) in Plasmodium falciparum using large-scale interaction network analysis and in-silico knock-out studies
Source: Malar J. 2015 Feb 8;14:70. doi: 10.1186/s12936-015-0562-1 (PMC4333160; doi:10.1186/s12936-015-0562-1)
Supplement: Additional file 21: Table S10. — Average Network Centrality parameters of 6 stage specific Networks. For each of the six life cycle stages six unique PPI network has been constructed analysed using graph theory principle. The table contains the average network centrality parameters for each of the stage. [file 12936_2015_562_MOESM21_ESM.doc]

|  | **Sporozoite** | **Merozoite** | **Trophozoite** | **RING** | **Schizont** | **Gametocyte** |
| --- | --- | --- | --- | --- | --- | --- |
| **Number of Nodes** | 617 | 438 | 1126 | 909 | 1132 | 1062 |
| **Number of edges** | 1458 | 1115 | 3074 | 2638 | 3079 | 2635 |
| **Number of Hubs** | 46 | 22 | 60 | 52 | 53 | 44 |
| **Diameter of the network** | 6 | 6 | 6 | 6 | 6 | 6 |
| **Average Distance** | 2.384 | 2.408 | 2.383 | 2.430 | 2.393 | 2.404 |
| **Average Clustering Coefficient** | 0.317 | 0.302 | 0.328 | 0.316 | 0.315 | 0.314 |
| **Average Eccentricity** | 0.257 | 0.253 | 0.245 | 0.251 | 0.277 | 0.248 |
| **Average Closeness** | 0.021 | 0.017 | 0.006 | 0.016 | 0.048 | 0.010 |
| **Average radiality** | 4.397 | 4.416 | 4.551 | 4.422 | 4.163 | 4.486 |
| **Average WienerIndex** | 2409.825 | 1011.858 | 2132.825 | 2659.777 | 1332.110 | 2640.506 |
| **Average Stress** | 1606.759 | 657.032 | 1456.064 | 1868.558 | 911.705 | 1782.821 |
| **Average S.P. Betweeness** | 1400.139 | 592.626 | 1238.753 | 1566.419 | 776.447 | 1543.293 |
| **Average degree** | 4.055 | 4.187 | 4.499 | 4.267 | 3.883 | 4.307 |
